# K_V_4.3 Expression Modulates Na_V_1.5 Sodium Current

**DOI:** 10.3389/fphys.2018.00178

**Published:** 2018-03-12

**Authors:** Vincent Portero, Ronald Wilders, Simona Casini, Flavien Charpentier, Arie O. Verkerk, Carol Ann Remme

**Affiliations:** ^1^Department of Experimental Cardiology, Academic Medical Center, Amsterdam, Netherlands; ^2^Department of Medical Biology, Academic Medical Center, Amsterdam, Netherlands; ^3^L'Institut du Thorax, INSERM, CNRS, Université de Nantes, Nantes, France

**Keywords:** transient outward current, sodium current, channels, action potential, myocyte, arrhythmias, computer simulation

## Abstract

In cardiomyocytes, the voltage-gated transient outward potassium current (I_to_) is responsible for the phase-1 repolarization of the action potential (AP). Gain-of-function mutations in *KCND3*, the gene encoding the I_to_ carrying K_V_4.3 channel, have been associated with Brugada syndrome (BrS). While the role of I_to_ in the pro-arrhythmic mechanism of BrS has been debated, recent studies have suggested that an increased I_to_ may directly affect cardiac conduction. However, the effects of an increased I_to_ on AP upstroke velocity or sodium current at the cellular level remain unknown. We here investigated the consequences of K_V_4.3 overexpression on Na_V_1.5 current and consequent sodium channel availability. We found that overexpression of K_V_4.3 protein in HEK293 cells stably expressing Na_V_1.5 (HEK293-Na_V_1.5 cells) significantly reduced Na_V_1.5 current density without affecting its kinetic properties. In addition, K_V_4.3 overexpression decreased AP upstroke velocity in HEK293-Na_V_1.5 cells, as measured with the alternating voltage/current clamp technique. These effects of K_V_4.3 could not be explained by alterations in total Na_V_1.5 protein expression. Using computer simulations employing a multicellular *in silico* model, we furthermore demonstrate that the experimentally observed increase in K_V_4.3 current and concurrent decrease in Na_V_1.5 current may result in a loss of conduction, underlining the potential functional relevance of our findings. This study gives the first proof of concept that K_V_4.3 directly impacts on Na_V_1.5 current. Future studies employing appropriate disease models should explore the potential electrophysiological implications in (patho)physiological conditions, including BrS associated with *KCND3* gain-of-function mutations.

## Introduction

The cardiac sodium current (I_Na_), generated by the *SCN5A*-encoded voltage-gated Na^+^ channel (Na_V_1.5) (Gellens et al., 1992), is responsible for the initial fast upstroke of the cardiac action potential (AP). It determines excitability of myocardial cells and ensures proper conduction of the electrical impulse within the heart. Consequently, Na_V_1.5 channel dysfunction may lead to conduction slowing, ventricular arrhythmias, and sudden cardiac death. In particular, *SCN5A* mutations leading to loss of sodium channel function are associated with isolated (progressive) conduction slowing or block (Schott et al., 1999), sick sinus syndrome (Benson et al., 2003), and Brugada syndrome (BrS) (Chen et al., 1998; Crotti et al., 2012; Le Scouarnec et al., 2015).

In cardiomyocytes, the voltage-gated transient outward K^+^ current (I_to_) is responsible for the phase-1 repolarization of the cardiac AP and thereby contributes to the refractory period and inotropic state of the myocardium. In human, I_to_ is generated by the K_V_4.3 channel, which is encoded by the gene *KCND3* (Niwa and Nerbonne, 2010). Gain-of-function mutations in *KCND3*, or its regulatory subunits, have also been associated with BrS (Delpón et al., 2008; You et al., 2015; Portero et al., 2016), giving rise to an ongoing discussion on the apparent role of I_to_ in the pro-arrhythmic mechanism of BrS (Wilde et al., 2010). Previous studies have suggested that an increased I_to_ may directly affect cardiac conduction due to a current-to-load mismatch during the depolarization process (Hoogendijk et al., 2010a,b). However, the effects of a gain-of-function of I_to_ on the AP upstroke velocity at the cellular level remain unknown. The characterization of various knock-out mouse models of α-subunits generating the fast component of I_to_ (*Kcnd3, Kcnd2*) confirmed the involvement of I_to_ in phase-1 repolarization, but its impact on AP upstroke velocity or I_Na_ density were not investigated (Niwa et al., 2008; Liu et al., 2015). We recently evaluated the effects of an *in silico* I_to_ injection on AP upstroke and repolarization velocity using the dynamic clamp technique (Verkerk et al., 2016). In that study, we observed a minimal effect (≈2%) of *in-silico* I_to_ injection on upstroke velocity but only when the injected current was large and rapidly activated at very negative potentials (around −50 mV). However, while an I_to_-like current does not appear to directly affect the fast depolarization, evidence is increasing that various ion channel proteins may interact and thus modulate each other's expression, function, or membrane trafficking (Balse and Boycott, 2017). For example, studies highlighted an interaction and co-regulation of the Kir2.1 and Na_V_1.5 proteins with a direct effect on their electrophysiological properties and thus important for cardiac excitability (Milstein et al., 2012). Moreover, a recent study demonstrated a direct interaction between the K_V_4.3 and hERG proteins, resulting in an increase in hERG current density upon co-expression of hERG with K_V_4.3 (Zhao et al., 2017). Considering these novel studies and the involvement of both I_Na_ and I_to_ in BrS, we hypothesized that the level of expression of K_V_4.3 may impact on I_Na_.

We addressed this hypothesis by evaluating the impact of K_V_4.3 protein overexpression on Na_V_1.5-based current and sodium channel availability in HEK293 cells stably expressing Na_V_1.5 (HEK293-Na_V_1.5). We show that an overexpression of K_V_4.3 channels leads to a reduction of Na_V_1.5 current density and lower AP upstroke velocity, as measured with the alternating voltage/current clamp (VC/CC) technique. These effects of K_V_4.3 overexpression could not be explained by alterations in total Na_V_1.5 protein expression. Computer simulations furthermore indicate that the experimentally observed decreased upstroke velocity is not directly due to an increase in the K_V_4.3-based I_to_, but instead a consequence of the K_V_4.3 protein itself. We also demonstrate that an increase in I_to_ as well as a decrease in I_Na_ can affect cardiac conduction and that a combination of both can lead to dramatic consequences, underlining the potential functional relevance of our findings.

## Materials and methods

### HEK293-Na_V_1.5 cell culture and transfection

To avoid artifacts due to co-transfections, we evaluated the effect of the overexpression of K_V_4.3 protein on Na_V_1.5 currents using a genetically modified cell line constitutively overexpressing the human Na_V_1.5 channel (van Bemmelen et al., 2004). HEK293 cells stably expressing hNa_V_1.5 (HEK293-Na_V_1.5, kindly provided by Drs. Hugues Abriel and Jean-Sébastien Rougier) were cultured in DMEM (Gibco) supplemented with 10% FBS (Biowest), L-glutamine (Gibco), penicillin-streptomycin (Gibco), and Zeomycin (Invitrogen) in a 5% CO_2_ incubator at 37°C. Cells were transfected at 70% confluency in 36.8 mm culture wells with 1 μg IRES-GFP or 1 μg *KCND3*-IRES-GFP cDNA using lipofectamine (Invitrogen, Carlsbad, USA). Both IRES-GFP and *KCND3*-IRES-GFP (human *KCND3* transcript reference: NM_004980.4) constructs contained the same M61 vector plasmid backbone (Addgene). Gene-transfer was monitored by means of green fluorescence from the IRES-GFP or *KCND3*-IRES-GFP bicistronic vector. Patch clamp experiments were performed on single fluorescent cells 2 days after transfection.

### Ventricular cell preparation

Animal procedures were performed in accordance with governmental and institutional guidelines for animal use in research and were approved by the Animal Experimental Committee of the Academic Medical Center, Amsterdam, The Netherlands. Single left ventricular myocytes were isolated from 3 to 5 months old FVB/N mice by enzymatic dissociation (Remme et al., 2006). Therefore, mice were anesthetized by an intraperitoneal injection of pentobarbital prior to cervical dislocation, after which the heart was excised, cannulated, and mounted on a Langendorff perfusion set-up. The hearts were perfused for 5 min with Tyrode's solution containing (in mM): 140 NaCl, 5.4 KCl, 1.8 CaCl_2_, 1 MgCl_2_, 5.5 glucose, 5 HEPES; pH 7.4 (NaOH). Subsequently, the heart was perfused for 8 min with a similar solution in which the calcium concentration was lowered to 1 μM, after which the enzyme Liberase Blendzyme type 4 (Roche; 0.05 mg/ml) and trypsin (Boehringer, 1 μl/ml of 2.5% solution) were added for 10 minutes. Single myocytes were obtained by gently triturating the digested tissue in the low calcium enzyme solution supplemented with bovine serum albumin (BSA, 50 mg/ml). Myocytes were washed twice in normal calcium Tyrode's solution and quiescent, rod-shaped cross-striated cells with smooth surface were selected for measurements.

### Western blot experiment for total Na_V_1.5 quantification

Forty-eight hours after transfection (1 μg IRES-GFP or 1 μg *KCND3*-IRES-GFP plasmid), HEK293-Na_V_1.5 cells were washed twice with PBS and lysed in PBS containing 0.1% Triton and complete mini EDTA-free protease inhibitor mixture tablet (Roche). Cell lysates were then sonicated twice for 10 s and centrifuged for 10 min at 8.000 rpm. The supernatant was quantified using the BCA kit and 40 μg of each separated cell lysate was loaded on a 4–20% gradient gel (Bio-Rad) after 5 min of denaturation at 60°C in Laemmli buffer. The RbαGFP antibody used for Western blotting was purchased from Santa Cruz Biotechnology, RbαCalnexin from Calbiochem, and RbαNa_V_1.5 from Sigma-Aldrich. Goat anti-rabbit horseradish peroxidase–conjugated secondary antibodies were purchased from GE Healthcare Life Science. Chemo luminescence signal was acquired with the ImageQuant LAS 4000 instrument. Band intensities were quantified using the public domain ImageJ software (USA National Institutes of Health). Total Na_V_1.5 protein expression protein was normalized to the calnexin signal.

### Electrophysiology

#### Data acquisition

Na_V_1.5 and K_V_4.3 currents and upstroke velocities (dV/dt) were measured in the whole-cell configuration of the patch-clamp technique using an Axopatch 200B amplifier (Molecular Devices Corporation, Sunnyvale, CA, USA) or a custom-made amplifier, capable of fast switching between voltage clamp (VC) and current clamp (CC) modes (Berecki et al., 2010). Voltage control, data acquisition, and analysis were accomplished using custom software. Potentials were corrected for the calculated liquid junction potentials (Barry and Lynch, 1991). Signals were low-pass filtered with a cut-off frequency of 5 kHz and digitized at 20, 4, and 40 kHz for Na_V_1.5, K_V_4.3, and upstroke velocities, respectively. Patch pipettes were pulled from borosilicate glass (Harvard Apparatus) and had resistances of ≈2.0 MΩ after filling with the solutions as indicated below. Series resistance was compensated by ≥80%. Cell membrane capacitance (C_m_) was calculated by dividing the time constant of the decay of the capacitive transient upon 5 mV hyperpolarizing voltage step from −40 mV by the series resistance. The average C_m_ was not significantly different between the IRES-GFP and K_V_4.3-IRES-GFP cells [IRES-GFP: 12.27 ± 0.76 pF (*n* = 36); K_V_4.3-IRES-GFP: 12.00 ± 0.99 pF (*n* = 37); mean±standard error of the mean (SEM)].

#### K_V_4.3 and Na_V_1.5 current measurements with conventional VC

Na_V_1.5 current was measured at room temperature (21°C) with patch pipettes containing (in mM): 60 CsCl, 50 aspartic acid, 11 EGTA, 1.0 CaCl_2_, 1.0 MgCl_2_, 5.0 Na_2_ATP, 10 HEPES, pH 7.2 (CsOH). Bath solution for Na_V_1.5 current measurements contained (in mM): 20 NaCl, 130 CsCl, 2 CaCl_2_, 1 MgCl_2_, 5 glucose, 10 HEPES, pH 7.4 (CsOH). K_V_4.3 current was measured at 37°C in Tyrode's solution with the aforementioned composition. Patch pipettes for K_V_4.3 current measurements were filled with solution containing (in mM): 125 K-gluc, 20 KCl, 5 NaCl, 1 MgCl_2_, 10 BAPTA, 5 MgATP, 10 HEPES, pH 7.2 (NMDG-OH).

Current density and gating properties were determined by means of custom voltage-clamp protocols as indicated in figure insets. Cycle lengths were 5 and 10 s for Na_V_1.5 and K_V_4.3 current, respectively. Both Na_V_1.5 and K_V_4.3 current densities were defined as the difference between peak and steady-state current, divided by C_m_. To determine the activation characteristics of Na_V_1.5 current, current-voltage (I-V) curves were corrected for driving force and normalized to maximum peak current. Steady-state activation and inactivation curves were fitted using the Boltzmann equation I/I_max_ = A/{1.0+exp[(V_1/2_−V)/k]} to determine V_1/2_ (membrane potential for the half-maximal (in)activation) and the slope factor k.

#### Upstroke velocity measurements using alternating VC/CC

The alternating VC/CC technique was used to measure Na_V_1.5 current-driven upstrokes at physiological temperature and Na^+^ concentrations, as described previously (Berecki et al., 2010). Patch pipette and bath solutions were similar to those used for K_V_4.3 current measurements (see above). HEK293-Na_V_1.5 cells and freshly isolated myocytes were voltage clamped at a holding potential of −85 mV, a value close to the resting membrane potential of working cardiomyocytes. Next, upstroke and repolarization were elucidated by switching for 20 ms to the CC mode of the patch clamp amplifier. Upstrokes were elicited by 1.2× threshold current pulses through the patch pipette at 0.5 Hz and the stimulus current was present during the entire 20 ms in CC mode. Maximal upstroke velocity (dV/dt_max_) during VC/CC, offline corrected for the contribution of stimulus current (I_stim_), served as an indicator of Na_V_1.5 current availability.

### Computer simulations

#### Numerical reconstruction of Na_V_1.5 and K_V_4.3 currents in HEK293 cells

Alternating VC/CC experiments in HEK293 cells expressing Na_V_1.5 and K_V_4.3 channels were simulated in a standard cell model with intracellular and extracellular sodium and potassium concentrations similar to those of the patch-clamp experiments. The cell model contained equations for I_Na_, I_to_, and I_stim_. The I_Na_ and I_to_ equations were taken from the human ventricular cell model by Ten Tusscher and Panfilov (2006) with the I_Na_ and I_to_ densities scaled by a factor of 1.8 and 6.0, respectively, to arrive at the experimentally observed values for the maximum upstroke velocity and repolarization velocity. I_stim_ was set to 6.5 pA/pF to ensure that the maximum upstroke velocity was reached at ≈4 ms after the stimulus onset as in the experiments. For numerical integration of the differential equations we applied a simple and efficient Euler-type integration scheme (Rush and Larsen, 1978) with a 1-μs time step.

#### Maximum upstroke velocity and repolarization velocity in murine left ventricular myocytes

The alternating VC/CC protocol of the experiments on single left ventricular myocytes was implemented in the CellML code (Cuellar et al., 2003) of the apical version of the mouse ventricular AP model by Bondarenko et al. (2004). The amplitude of the 20 ms stimulus current was set to 10 pA/pF, which made the AP upstroke occur at ≈4 ms after the stimulus onset. The maximum upstroke velocity and repolarization velocity were determined from the time derivative of the action potential trace. The CellML code was edited and run in version 0.9.31.1409 of the Windows based Cellular Open Resource (COR) environment (Garny et al., 2003) with its standard CVODE integrator.

#### Action potential transfer in human left ventricular myocytes

AP transfer was studied in a one-dimensional strand of human left ventricular myocytes. The strand consisted of 80 longitudinally coupled cells, each described by the Ten Tusscher and Panfilov model of a single human ventricular myocyte (Ten Tusscher and Panfilov, 2006). The intercellular coupling conductance was set to 6 nS and the myoplasmic resistivity was set to 150 Ω·cm. The first cell of the strand was stimulated at a rate of 1 Hz with a 2-ms, ≈20% supra-threshold stimulus current with an amplitude of 3.2 nA. As in other studies (Shaw and Rudy, 1997; Thomas et al., 2003; Wilders, 2012), the entire cell length (of 74 μm) was used as the spatial discretization element, with elements connected by the lumped myoplasmic resistance (calculated from the myocyte dimensions and the myoplasmic resistivity of 150 Ω·cm) and gap junctional resistance. At the selected gap junctional conductance of 6 nS, this lumped resistance was almost completely determined by the gap junctional resistance (Wilders, 2012). The aforementioned Euler-type integration scheme was used for numerical integration.

### Statistics

Data are expressed as mean±SEM. Mann-Whitney Rank Sum test, Student *t*-test, or Two-Way Repeated Measures ANOVA followed by pairwise comparison using the Student-Newman-Keuls test were used when appropriate. The level of statistical significance was set to *p* < 0.05.

## Results

### Characterization of K_V_4.3 current in HEK293 cells stably expressing Na_V_1.5

**Figure 1:**
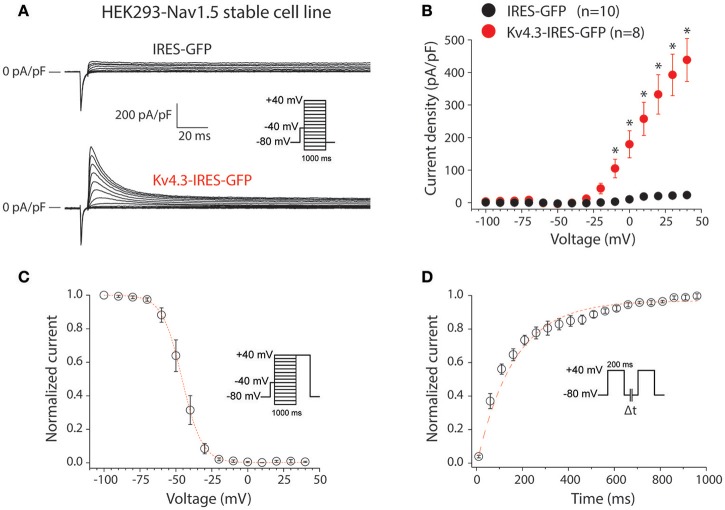
K_V_4.3 currents in HEK293-Na_V_1.5 cells transfected with *KCND3***. (A)** Typical current traces in response to voltage clamp steps to test potentials ranging from −100 to 40 mV recorded from HEK293 cells stably expressing Na_V_1.5 (HEK293-Na_V_1.5) and transfected with IRES-GFP (top) or *KCND3*-IRES-GFP (bottom). Inset: voltage clamp protocol used. **(B)** Current-voltage (I-V) relationships of the peak outward current in *KCND3*-IRES-GFP and IRES-GFP transfected cells. Asterisks denote *p* < 0.05. **(C)** Voltage-dependency of K_V_4.3 inactivation in K_V_4.3-IRES-GFP cells (*n* = 8). The dotted line is the Boltzmann fit to the average data. Inset: voltage clamp protocol used. **(D)** Average recovery from inactivation curve measured with two subsequent 200-ms pulses from −80 to +40 mV with variable inter-pulse durations (Δt) of 1–1,000-ms (*n* = 4). Inset: voltage clamp protocol used. The red dashed line is a mono-exponential fit of the average data.

First, we characterized the K_V_4.3-driven current resulting from transfection of the *KCND3*-IRES-GFP plasmid in HEK293 cells stably expressing Na_V_1.5 (HEK293-Na_V_1.5). Figure 1A shows representative currents measured between −100 to 40 mV (with 10 mV increment) during 1-s voltage clamp steps from a holding potential of −80 mV in a HEK293-Na_V_1.5 cell transfected with either IRES-GFP (top) or *KCND3*-IRES-GFP (bottom). A prepulse of 5 ms to −40 mV was applied to activate and inactivate the Na_V_1.5-based sodium current present in these HEK293-Na_V_1.5 cells (Figure 1A, inset). Similar to previous reports, cells transfected with IRES-GFP showed a small endogenous current upon depolarization (Figure 1A, top) (Niwa et al., 2008). In contrast, cells transfected with *KCND3*-IRES-GFP displayed a large K_V_4.3 current which started to activate around −40 mV and increased in amplitude upon further depolarization due to enhanced activation and increased K^+^ driving force (Figure 1A, bottom) (Giles and van Ginneken, 1985). Figure 1B shows the average I-V relationships of the K_V_4.3 current, which was defined as the difference between the peak and steady-state current. Its density was defined as the current amplitude divided by the membrane capacitance (C_m_), amounting to ≈450 pA/pF at 40 mV in the *KCND3*-IRES-GFP transfected HEK293-Na_V_1.5 cells, and >20 times smaller in the IRES-GFP transfected cells. Figure 1C shows the voltage-dependency of inactivation for the K_V_4.3 current in the cells transfected with *KCND3*-IRES-GFP, measured using a two-pulse protocol with a 1-s prepulse to a potential between −100 and 40 mV followed by a 500-ms test pulse to 40 mV, demonstrating that the K_V_4.3 current is fully available at −70 mV and more negative potentials. The V_1/2_ and k of the voltage-dependency of inactivation were −45.9 ± 2.6 and −5.6 ± 0.4 mV, respectively. Figure 1D shows the recovery from inactivation in *KCND3*-IRES-GFP transfected HEK293-Na_V_1.5 cells analyzed with 200-ms pulses to 40 mV with variable inter-pulse intervals. The time constant of recovery from inactivation, analyzed with a mono-exponential fit (Figure 1D, dashed line) was 160 ± 29 ms, demonstrating that recovery from inactivation was completed with an inter-pulse interval of 1 s and longer. The K_V_4.3 current biophysical properties are summarized in Table S1. These data indicate that overexpression of *KCND3*-IRES-GFP in HEK293-Na_V_1.5 cells results in a large I_to_, confirming the functional expression of K_V_4.3 channels at the cell membrane.

### K_V_4.3 expression reduces Na_V_1.5 current in HEK293-Na_V_1.5 cells

**Figure 2:**
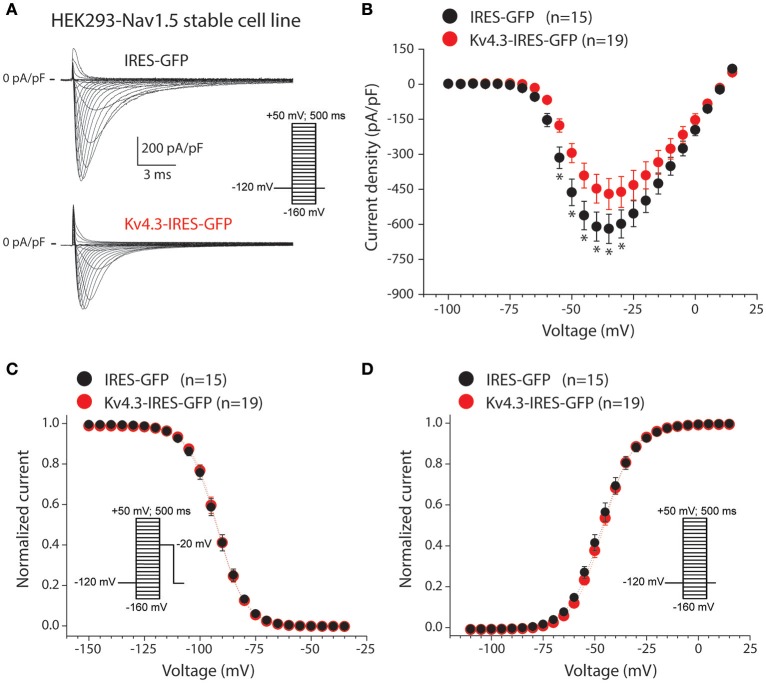
K_V_4.3 overexpression reduces Na_V_1.5 currents**. (A)** Typical Na_V_1.5 current traces in response to 500-ms depolarizing voltage steps from a holding potential of −120 mV to test potentials ranging from −160 to 50 mV in IRES-GFP (top) and *KCND3*-IRES-GFP (bottom) transfected HEK293-Na_V_1.5 cells. Inset: voltage clamp protocol used. **(B)** Average I-V relationships of the Na_V_1.5 peak current in IRES-GFP and *KCND3*-IRES-GFP transfected cells. Asterisks denote *p* < 0.05. **(C,D)** Average steady-state inactivation **(C)** and activation **(D)** curves. Insets: voltage clamp protocol used. Voltage-dependency of inactivation was measured using a two-pulse protocol where a 500-ms conditioning prepulse to membrane potentials between −160 and 50 mV (to induce steady-state inactivation), was followed by a 50-ms test pulse to −20 mV. Voltage-dependency of activation was measured using the same protocol described as described in **(A)**.

**Figure 3:**
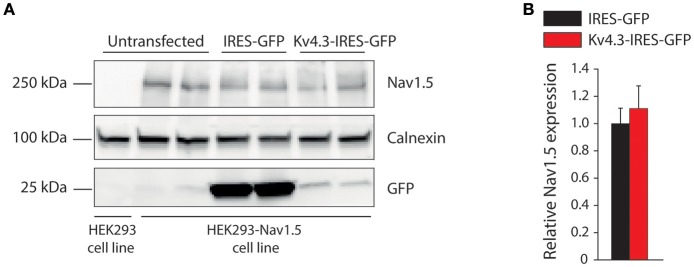
Na_V_1.5 total expression is not affected by K_V_4.3 overexpression**. (A)** Representative Western blots of Na_V_1.5, calnexin and GFP protein expression in HEK293-Na_V_1.5 cells. Data from IRES-GFP and *KCND3*-IRES-GFP transfected cells as well as untransfected cells. Total lysate of HEK293 cells lacking the Na_V_1.5 overexpression cassette (leftmost lane) was used as negative control for Na_V_1.5 antibody specificity. **(B)** Average total Na_V_1.5 expression normalized to calnexin protein expression in *KCND3*-IRES-GFP transfected HEK293-Na_V_1.5 cells, compared to IRES-GFP transfected HEK293-Na_V_1.5 cells (*n* = 6, 3 different blots).

Secondly, we characterized the effects of K_V_4.3 overexpression on Na_V_1.5-based current in HEK293-Na_V_1.5 cells. Figure 2A shows representative Na_V_1.5 current activated by 500-ms depolarizing voltage clamp steps of 5 mV increment from a holding potential of −120 mV in a IRES-GFP or *KCND3*-IRES-GFP transfected HEK293-Na_V_1.5 cell. In both conditions, Na_V_1.5 current started to activate around −60 mV, peaked around −30 mV, and subsequently decreased in amplitude due to the reduction in Na^+^ driving force. As shown in the average I-V relationships in Figure 2B, Na_V_1.5 current density was significantly smaller in the *KCND3*-IRES-GFP transfected cells compared to IRES-GFP transfected cells. For example, at −40 mV Na_V_1.5 current density was −609 ± 62 and −447 ± 61 pA/pF (*p* < 0.05; pairwise comparison using a Student-Newman-Keuls test following two-way repeated measures ANOVA) in IRES-GFP and *KCND3*-IRES-GFP transfected HEK293-Na_V_1.5 cells, respectively, indicating a decrease in Na_V_1.5 current density by ≈25% due to K_V_4.3 overexpression. Next, we determined whether the Na_V_1.5 current density decrease was accompanied by gating property changes. Because the current decay could not be reliably fit to a 2-exponential function, the time course of inactivation was instead determined by analyzing the time required for 50% of current decay to occur (t_50%_) at −30 mV (Remme et al., 2006). t_50%_ did not differ significantly between IRES-GFP and *KCND3*-IRES-GFP transfected cells [0.88 ± 0.06 ms (*n* = 15) vs. 0.84 ± 0.03 ms (*n* = 19), respectively, *p* = 0.41; Student's *t*-test]. For determining the voltage-dependency of activation for IRES-GFP and *KCND3*-IRES-GFP transfected cells, I-V relationships, as shown in Figure 2B, were first corrected for the Na^+^ driving force. Of note, the reversal potential of Na_V_1.5 current calculated using the Nernst equation is evaluated at +17.58 mV, which is in line with the sodium current recordings represented in Figure 2A. Next, currents were normalized to their maximal amplitude and the curves were fitted to a Boltzmann distribution curve. Figure 2C shows overlapping curves of voltage-dependency of inactivation. The latter curves were constructed by normalizing currents to their maximal current during the voltage clamp step to −20 mV. Similarly, Figure 2D shows overlapping curves of voltage-dependency of activation for IRES-GFP and *KCND3*-IRES-GFP transfected cells. These data demonstrate that neither the voltage-dependency of activation nor the voltage-dependency of inactivation of Na_V_1.5 current were affected by K_V_4.3 expression. The Na_V_1.5 current biophysical properties are summarized in Table S1. Western blot analysis showed no differences in Na_V_1.5 protein expression levels between HEK293-Na_V_1.5 cells with overexpression of *KCND3*-IRES-GFP or GFP (Figure 3; original images provided in the Supplementary Material, Figure S1). Thus, our data demonstrate that K_V_4.3 expression reduces Na_V_1.5 current without affecting its gating properties or the Na_V_1.5 expression level.

### K_V_4.3 expression reduces action potential upstroke velocity

**Figure 4:**
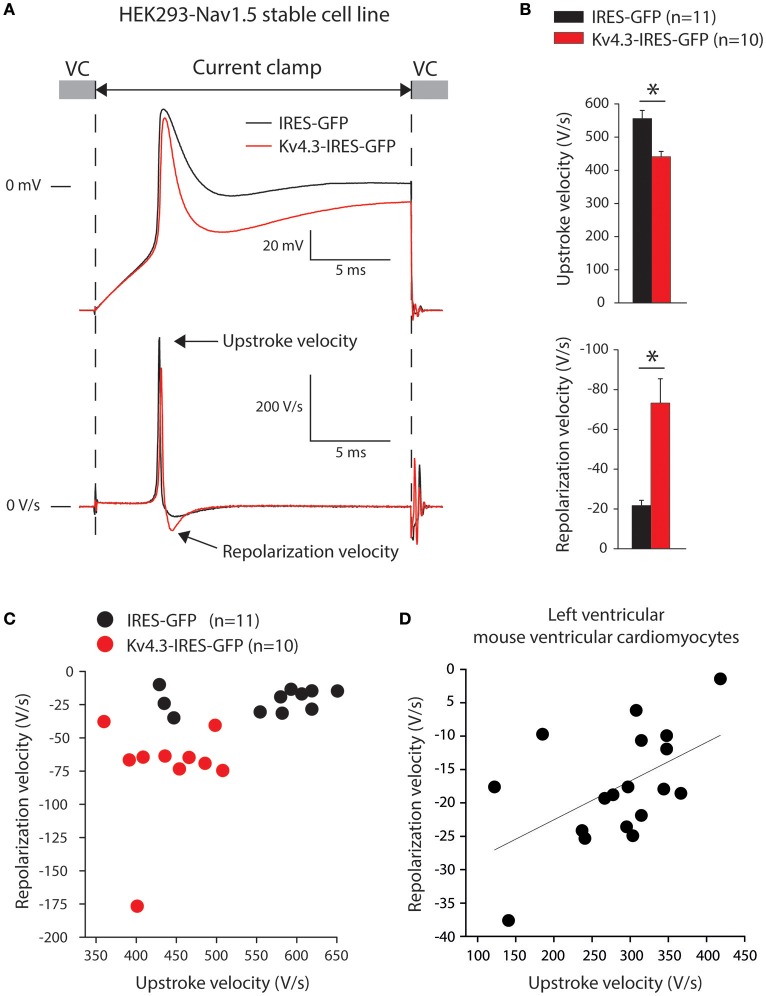
K_V_4.3 overexpression reduces upstroke velocities**. (A)** Typical examples of upstrokes in *KCND3*-IRES-GFP and IRES-GFP transfected HEK293-Na_V_1.5 cells measured with the alternating voltage/current clamp (VC/CC) technique (top) and time derivatives of these upstrokes (bottom). Arrows indicate the maximum AP upstroke and repolarization velocities. **(B)** Average AP upstroke (top) and repolarization velocities (bottom). Asterisks denote *p* < 0.05. **(C)** Relationship of upstroke and repolarization velocities in HEK293-Na_V_1.5 cells. **(D)** Relationship of AP upstroke and repolarization velocities in murine isolated left ventricular myocytes. The solid line represents the linear fit (Pearson's coefficient: *r* = 0.53).

Thirdly, we assessed upstroke velocities in the HEK293-Na_V_1.5 cells, transfected with either IRES-GFP or *KCND3*-IRES-GFP plasmids. Noteworthy, HEK293 cells expressing Na_V_1.5 display fast depolarizations upon switching from voltage clamp (VC) to current clamp (CC) due to Na^+^ channel activation as previously shown (Berecki et al., 2010; Verkerk et al., 2016; Lieve et al., 2017). Thus, the alternating VC/CC technique allows for a more dynamic assessment of Na_V_1.5 current as compared to measurements in VC configuration, in the setting of more physiological temperature and Na^+^ gradients across the membrane. Figure 4A shows typical upstrokes (top) and their time derivatives (bottom) measured upon switching from a holding potential of −85 mV in VC to CC for 20-ms. Upstrokes were evoked at 0.5 Hz, a stimulus frequency at which K_V_4.3 current is fully available (cf. Figure 1D). Figure 4B summarizes the maximum upstroke velocities (top) and maximum repolarization velocities (bottom). On average, the maximum upstroke velocity of *KCND3*-IRES-GFP transfected cells was 21% lower than that of IRES-GFP transfected cells (441 ± 17 vs. 556 ± 24 V/s; *p* < 0.05). Both cell types displayed a repolarization phase following the upstroke (Figure 4A) with a significantly larger maximum velocity (73 ± 13 vs. 22 ± 3 V/s) in *KCND3*-IRES-GFP as compared to IRES-GFP transfected cells (Figure 4B, bottom). In Figure 4C, we plotted for each cell its maximum repolarization velocity vs. its maximum upstroke velocity, further demonstrating an inverse relationship between Na_V_1.5-induced depolarization and K_V_4.3-induced repolarization velocities: K_V_4.3 expression increases repolarization velocity, but reduces upstroke velocity.

### Relation between repolarization and depolarization in ventricular myocytes of mice

The above presented data indicate that K_V_4.3 (over)expression decreases Na_V_1.5 current (Figure 2B) and upstroke velocity (Figure 4B), resulting in an inverse relationship between the maximum velocities of depolarization and repolarization (Figure 4C). Next, we determined whether a relationship between repolarization and depolarization also exists in cardiomyocytes. Therefore, we re-analyzed data of alternating VC/CC measurements in murine isolated left ventricular myocytes performed for a previous study (Remme et al., 2006). In mouse ventricular myocytes, the maximum upstroke and repolarization velocities were 285 ± 19 and −18 ± 2 V/s (*n* = 18), respectively. In Figure 4D, we plotted for each cell its maximum repolarization velocity vs. its maximum upstroke velocity, demonstrating an inverse relationship, similar to our experiments on HEK293 cells (Figure 4C).

### Computer simulations

**Figure 5:**
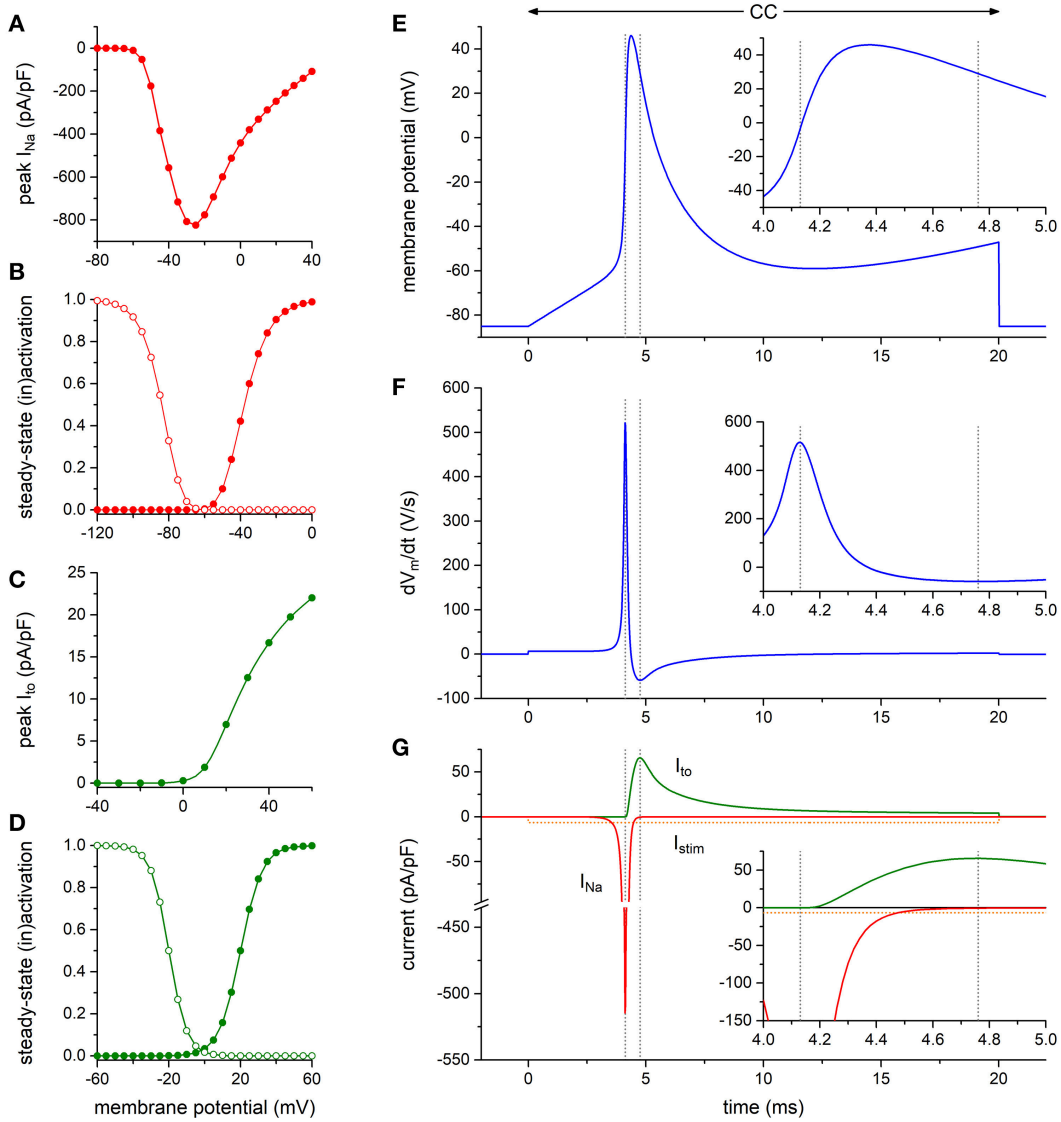
Numerical reconstruction of Na_V_1.5 and K_V_4.3 currents in alternating VC/CC experiments**. (A–D)** Characteristics of the simulated hNa_V_1.5 driven fast sodium current (I_Na_) and hK_V_4.3 driven transient outward current (I_to_). **(A)** I_Na_ peak current as obtained with the voltage clamp protocol of Figure 2. **(B)** I_Na_ steady-state activation and inactivation curves (closed and open symbols, respectively) as obtained with the voltage clamp protocol of Figure 2. **(C)** I_to_ peak current as obtained with the voltage clamp protocol of Figure 1. **(D)** I_to_ steady-state activation and inactivation curves (closed and open symbols, respectively). **(E–G)** Reconstruction of the alternating VC/CC experiment. **(E)** Membrane potential (V_m_) during the 20-ms current clamp (CC) phase. Inset: V_m_ on an expanded time scale. **(F)** Time derivative of the membrane potential trace of **(E)**. Inset: dV_m_/dt on an expanded time scale. **(G)** Individual time courses of I_Na_, I_to_, and the 20-ms inward stimulus current (I_stim_). Note the axis break. Inset: I_Na_, I_to_, and I_stim_ on an expanded time scale. The vertical dashed lines in **(E–G)** indicate the time of the maximum upstroke velocity (left lines) and the time of the maximum repolarization velocity (right lines). Alternating VC/CC protocol as in the patch-clamp experiments on HEK293-Na_V_1.5 cells.

To assess the functional relevance of the aforementioned findings, we first carried out computer simulations to explore whether the presence of a K_V_4.3-based I_to_ *per se* may affect the maximum upstroke velocity of the HEK293-Na_V_1.5 cells. To this end, we constructed a model of a HEK293 cell expressing both I_Na_ and I_to_ channels to further explore the findings of the alternating VC/CC experiment of Figures 4A,B. The I_Na_ and I_to_ equations were taken from the human ventricular cell model by Ten Tusscher and Panfilov (2006). The characteristics of the simulated I_Na_ and I_to_ are shown in Figures 5A–D (and also in Figures S2, S3). As illustrated in Figures 5E–G, I_to_ is effectively zero at the moment of maximum upstroke velocity, indicating that this maximum upstroke velocity is fully determined by I_Na_. Conversely, I_Na_ is almost zero at the moment of maximum repolarization velocity. Accordingly, there is no direct contribution of I_Na_ to the maximum repolarization velocity, although it must be noted that I_Na_ may still affect repolarization velocity through its effect on AP shape. If the voltage-dependency of the I_to_ channels is shifted by −30 mV, thus letting them start to activate near −40 mV, I_to_ is still effectively zero at the moment of maximum upstroke velocity (data not shown).

**Figure 6:**
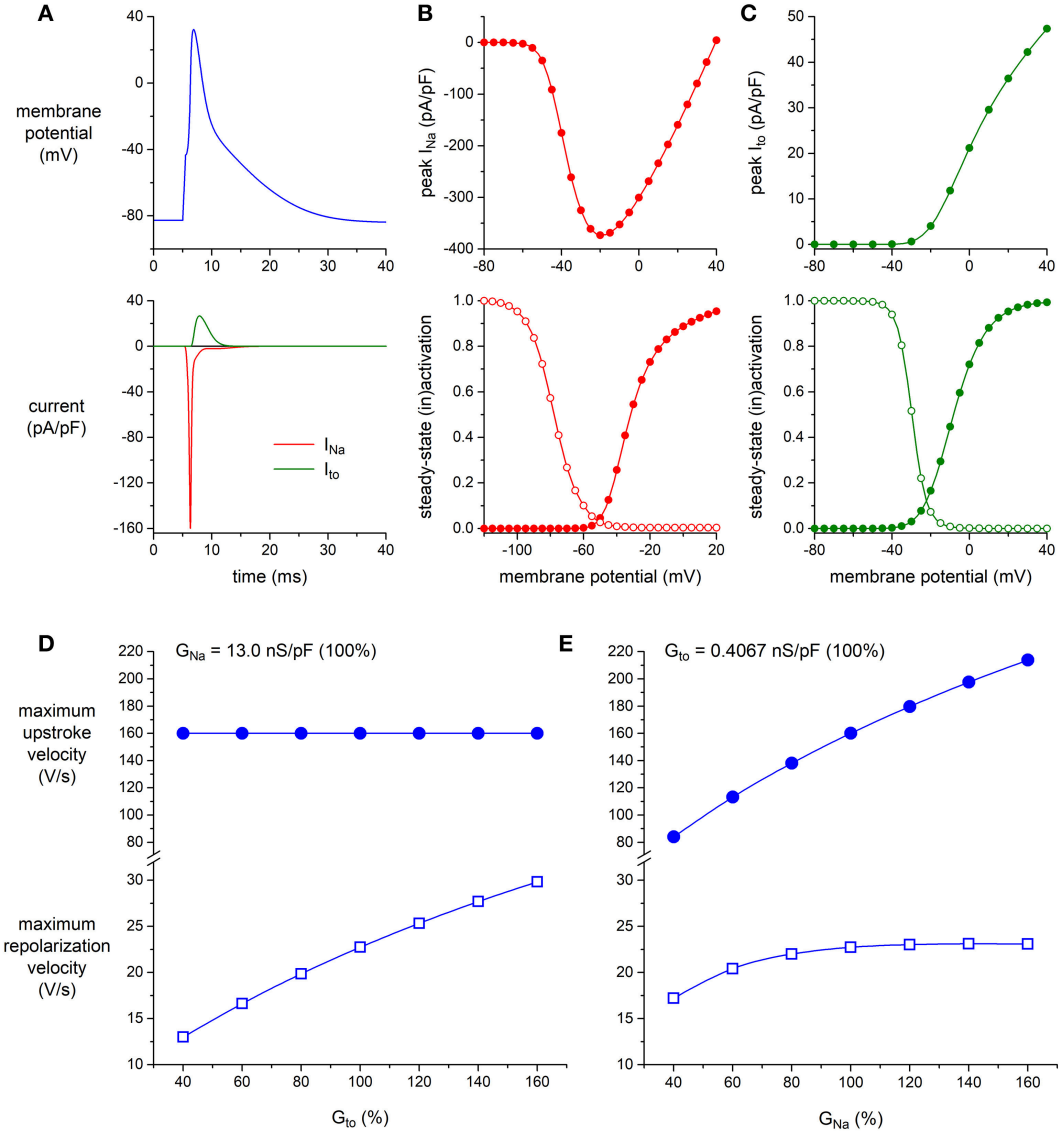
Effect of I_Na_ and I_to_ density on maximum upstroke and repolarization velocity in simulated murine left ventricular myocytes. **(A–C)** Characteristics of the computer model of apical mouse ventricular myocytes (Bondarenko et al., 2004) used in the simulations. **(A)** Action potential (top) and associated I_Na_ and I_to_ (bottom). **(B)** I_Na_ peak current (top) and steady-state activation and inactivation curves (bottom; closed and open symbols, respectively), as obtained with the voltage clamp protocol of Figure 2. **(C)** I_to_ peak current (top) and steady-state activation and inactivation curves (bottom; closed and open symbols, respectively), as obtained with the voltage clamp protocol of Figure 1. **(D,E)** Maximum upstroke velocity and maximum repolarization velocity as obtained in a reconstruction of the alternating VC/CC experiment. **(D)** Maximum upstroke velocity (filled symbols) and maximum repolarization velocity (open symbols) as a function of I_to_ conductance (G_to_) at a constant value of I_Na_ conductance (G_Na_). **(E)** Maximum upstroke velocity and maximum repolarization velocity as a function of G_Na_ at a constant value of G_to_. Alternating VC/CC protocol as in the patch-clamp experiments on murine left ventricular myocytes.

Next, we established to which extent the K_V_4.3-based I_to_ and the Na_V_1.5-based I_Na_ *per se* modulate the maximum upstroke velocity and repolarization velocity of murine left ventricular myocytes. To this end, we ran computer simulations of the alternating VC/CC experiment on mouse ventricular myocytes of Figure 4D using the apical version of the mouse ventricular AP model by Bondarenko et al. (2004), in which we increased or decreased the density of either I_to_ or I_Na_. The characteristics of I_Na_ and I_to_ of the Bondarenko et al. (2004) model are shown in Figures 6A–C (and also in Figures S2, S3). As shown in Figure 6D, and as expected from Figure 5, the maximum upstroke velocity is independent of I_to_ conductance (G_to_), whereas G_to_ is an important determinant of repolarization velocity. The maximum repolarization velocity increases almost linearly with G_to_ (Figure 6D, open squares), albeit not in a 1:1 fashion—a four times increase in G_to_ (from 40 to 160% of its control value) results in a 2.3 times increase in maximum repolarization velocity, which can be explained by accompanying changes in AP shape (and thus in channel activation and inactivation) as well as the presence of other membrane currents. As shown in Figure 6E, the maximum upstroke velocity is strongly dependent on I_Na_ conductance (G_Na_), whereas repolarization velocity is only substantially affected by G_Na_ at lower values of G_Na_. At these lower values, the AP reaches a considerably lower peak (data not shown), which in turn results in less activation of I_to_ channels as well as a lower driving force for these channels. At control or higher values of G_Na_, repolarization velocity is not notably dependent on G_Na_.

**Figure 7:**
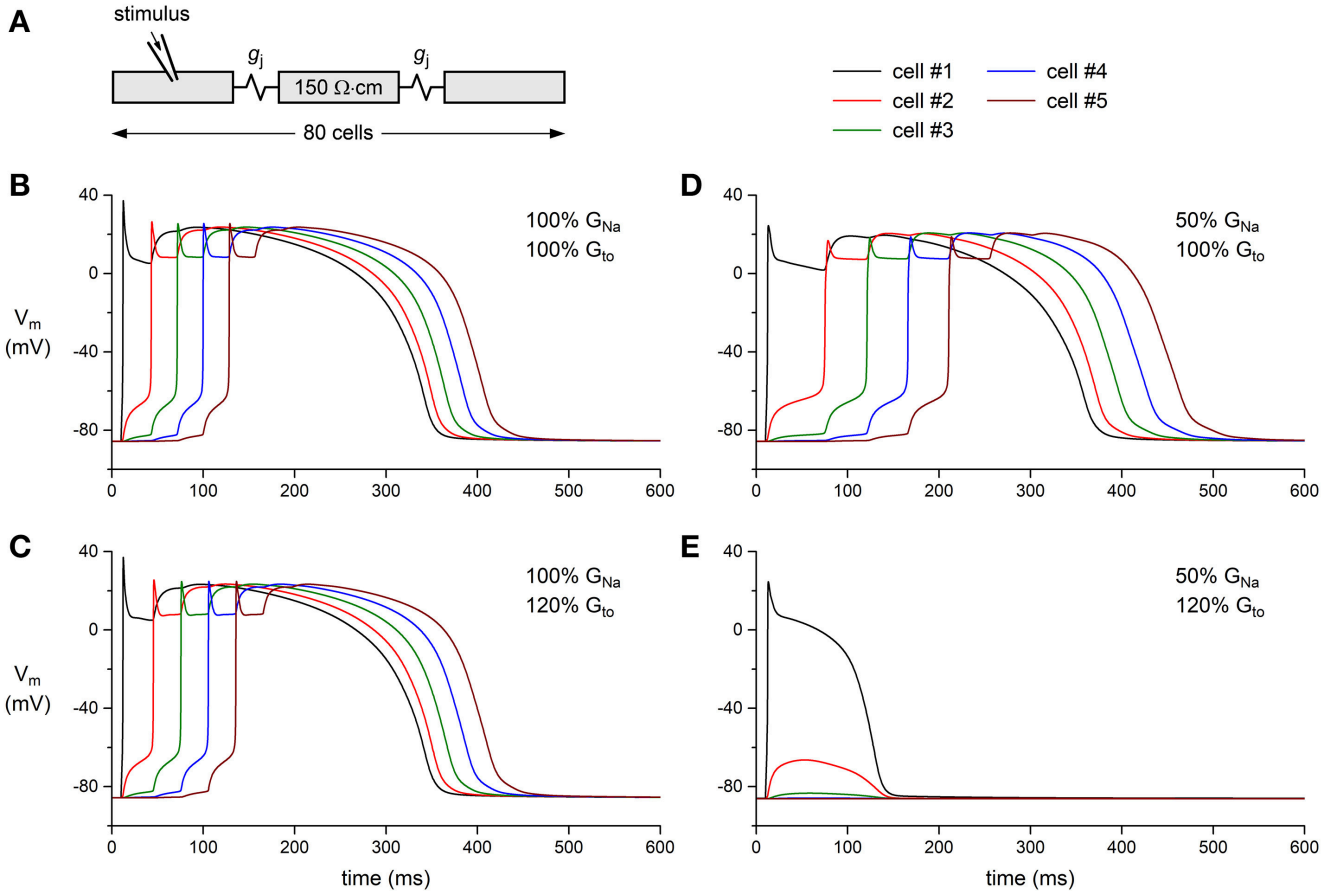
Effect of I_Na_ and I_to_ density on action potential propagation in a simulated strand of human left ventricular myocytes. **(A)** Geometry of the strand. The intercellular coupling conductance (g_j_) was set to 6 nS and the myoplasmic resistivity was set to 150 Ω·cm. **(B–E)** Action potential propagation in the strand as a function of I_Na_ conductance (G_Na_) and I_to_ conductance (G_to_). **(B)** Control conditions: G_Na_ and G_to_ both set to 100% of their control value. **(C)** Slight slowing of action potential propagation upon an increase in G_to_ to 120% of its control value. **(D)** Slowing of action potential propagation upon a decrease in G_Na_ to 50% of its control value. **(E)** Failure of action potential propagation upon a concomitant decrease in G_Na_ and increase in G_to_.

Finally, we tested whether a decrease in G_Na_ can result in a loss of conduction in the presence of a concomitant increase in G_to_. This was studied in a one-dimensional strand of poorly coupled human left ventricular myocytes, as illustrated in Figure 7A. Under control conditions (100% G_Na_ and 100% G_to_), the stimulated leftmost cell of the strand was able to drive its neighboring cells (Figure 7B). This was also observed upon a 20% increase in G_to_ or a 50% decrease in G_Na_ (Figures 7C,D, respectively). The main effect of the decrease in G_Na_ was a slowing of conduction (cf. Table 1). However, the introduction of a concomitant 20% increase in G_to_ led to loss of conduction: an action potential was elicited in the first cell of the strand, but this cell was unable to transfer this action potential to its neighboring cells (Figure 7E). Thus, a simultaneous increase in I_to_ and decrease in I_Na_ in the setting of a compromised cellular coupling, as may occur on a microscopic scale in BrS patients, may result in severe conduction alteration.

**Table 1:** Action potential parameters and propagation in a strand of human left ventricular myocytes.

**Settings**	**APA (mV)**	**(dV_*m*_/dt)_max_ (V/s)**	**APD_90_ (ms)**	**CV (cm/s)**
100% G_Na_, 100% G_to_	111.3	223.5	275.6	0.260
100% G_Na_, 120% G_to_	110.5	217.1	272.1	0.245
50% G_Na_, 100% G_to_	104.6	79.0	248.1	0.163

*Data obtained from computer simulations with an intercellular coupling conductance of 6 nS. APA, action potential amplitude; (dV_m_/dt)_max_, maximum upstroke velocity; APD_90_, action potential duration at 90% repolarization; CV, conduction velocity in the strand. Action potential parameters for cells in the middle of the strand*.

## Discussion

In the present study, we report for the first time a direct effect of K_V_4.3 expression on Na_V_1.5 current and consequent sodium channel availability. We found that overexpression of K_V_4.3 protein reduces Na_V_1.5 current density (Figure 2) without affecting total Na_V_1.5 protein expression. Moreover, K_V_4.3 overexpression decreased the upstroke velocity, which was not due to a direct effect of the current generated by K_V_4.3. These findings suggest that the observed effects of K_V_4.3 on Na_V_1.5 are due to a functional interaction between the two channel proteins rather than an electrophysiological interference. The physiological relevance of our findings was demonstrated in a multicellular *in silico* model where we found that an I_to_ increase and concomitant I_Na_ decrease is capable of impairing conduction (Figure 7).

### Decreased AP upstroke velocity secondary to K_V_4.3 overexpression

Using the alternating VC/CC technique, we observed in HEK293 cells stably expressing Na_V_1.5 a decrease in upstroke velocity secondary to overexpression of K_V_4.3. Upstroke velocity decreased to a similar extent as Na_V_1.5 sodium current density (≈25%) in cells overexpressing K_V_4.3 compared to cells overexpressing only GFP. Although the alternating VC/CC technique is not commonly used in cardiac electrophysiology, it allows detailed I_Na_ studies in a dynamic fashion under close-to-physiological conditions as occurring during cardiac action potentials (Berecki et al., 2010). Previously it has been used to demonstrate changes in I_Na_ during heart failure (Berecki et al., 2010), changes in intracellular Ca^2+^ concentration (Casini et al., 2009), and in the setting of *SCN5A* mutations (Remme et al., 2006; Lieve et al., 2017). Using dynamic clamp, we have recently assessed the effect of an I_to_-like current on the upstroke velocity in HEK293 cells transiently overexpressing Na_V_1.5 channels. That study revealed only a minor influence of I_to_, if any, on upstroke velocity (Verkerk et al., 2016). Here, we confirmed these results using computer simulations based on the current densities recorded (Figure 5). The I_to_ current is not yet present at the time of the maximum upstroke velocity confirming that I_to_ does not affect the upstroke velocity in single cells. Moreover, no correlation between I_to_ density and AP upstroke velocity was observed during simulations of the electrical activity of cardiomyocytes (Figure 6). Thus, the observed K_V_4.3-induced decrease in upstroke velocity is not a consequence of an increased K_V_4.3 current.

In freshly isolated left ventricular myocytes of mice, we found an inverse relationship between the maximum upstroke velocity and repolarization velocity (Figure 4D). It is tempting to speculate that such a relationship is due to effects of K_V_4.3 expression, but this observation might also be due to different currents or transcription gradient of both *Kcnd3* and *Scn5a* through the myocardial wall. Nevertheless, it indicates that depolarization and repolarization are not independent factors. Future overexpression or knock-down experiments in myocytes are needed to provide further insight into the potential relevance of our observation.

### Modulation of Na_V_1.5 current by K_V_4.3 expression

We found that K_V_4.3 overexpression resulted in a significantly decreased Na_V_1.5 current density, while neither current kinetics nor total Na_V_1.5 protein expression were affected. Several studies have previously reported interactions and consequent inter-relationships between sodium and potassium channels, or their associated subunits, leading to electrophysiological modifications of either current (Hu et al., 2012; Milstein et al., 2012; Matamoros et al., 2016; Utrilla et al., 2017). For instance, Kir2.1 and Na_V_1.5 channels when co-expressed exert a synergic effect on current density due to a distinct trafficking process as compared to when these channels are expressed separately (Utrilla et al., 2017). Concerning the direct interaction of K_V_4.3 and Na_V_1.5, a mutation in the *SCN1B* gene encoding the cardiac sodium channel β1 subunit has been found in BrS and sudden infant death syndrome patients (Hu et al., 2012). This specific *SCN1B* mutation led to a decreased I_Na_ together with an increased I_to_, suggesting that Na_v_1.5 may physically interact with K_V_4.3 via β1 to form a macromolecular complex. In neurons, both sodium and potassium channels mainly localize at the axon initial segment (AIS), a subcellular region involved in AP genesis. Ion channel recruitment to the AIS is highly dependent on the cytoskeleton and associated proteins which densely populate this region (Brachet et al., 2010; Leterrier et al., 2011; Vacher et al., 2011). A comparable structure is observed at the intercalated discs in adult cardiomyocytes, a subcellular region also characterized by a high density of sodium channels (Agullo-Pascual et al., 2014; Marsman et al., 2014; El Refaey et al., 2017). Our findings, together with existing knowledge, may suggest a potential subcellular co-localization of K_V_4.3 and Na_V_1.5 in cardiomyocytes and a possible explanation for the co-regulation of channel function. Further studies should aim at unraveling the molecular mechanism involved in our observation. A recent work also highlighted the synergic impact of K_V_4.3 expression on hERG channels in HEK293 cells (Zhao et al., 2017). This study together with our findings strongly suggest that the overexpression or knock-down of K_V_4.3 in cardiomyocytes may lead to a complex modulation of ion channels initially seen as unrelated including Na_V_1.5, hERG, and Kir2.1 channels.

### Potential (patho)physiological implications

Our computer simulations show that a concomitant increase in I_to_ and decrease in I_Na_ are capable of significantly affecting cardiac conduction. Our current observations may thus be particularly relevant in the phenotypical explanation of BrS patients presenting with mutations in the *KCND3* gene or in genes encoding K_V_4.3 associated subunits leading to gain-of-function through an increased protein stability or membrane trafficking of K_V_4.3 channels. In this context, an increased I_to_ current could be associated with a simultaneous decrease in I_Na_ and consequent conduction slowing (Kucera et al., 2017). Such an effect is likely even more prominent within the subepicardium, a region with relatively low I_Na_ and high I_to_. This may be of particular relevance in the right ventricular subepicardium and right ventricular outflow tract, where fractionated signals indicative of slowed conduction are often observed in BrS patients (Nademanee et al., 2011). Importantly, concomitant cardiac structural abnormalities, for example age-dependent development of fibrosis (Coronel et al., 2005), may further compromise conduction and hence unmask the impact of increased I_to_ (Hoogendijk et al., 2010a). Moreover, one may speculate that the known gender differences in I_to_ magnitude may also contribute to the male preponderance for BrS via an indirect effect on I_Na_ (Di Diego et al., 2002).

### Limitations

While we clearly demonstrated that K_V_4.3 is capable of decreasing Na_V_1.5 current density, extrapolation to physiological cardiomyocyte conditions must be done with some caution. The upstroke velocities measured in our experiments are in the same range of values as recorded in cardiomyocytes (Berecki et al., 2010; Veerman et al., 2017). However, *KCND3* overexpression resulted in K_V_4.3 currents larger than the I_to_ previously reported in various animal species and human ventricular cardiomyocytes (Niwa and Nerbonne, 2010). Consequently, the K_V_4.3-induced ≈25% reduction of Na_V_1.5 current density and upstroke velocity reported in this study is likely smaller in animal species and human. Moreover, our experiments in HEK293 cells were performed without β subunits of either Na_V_1.5 or K_V_4.3. We therefore cannot exclude a supplementary complexity of Na_V_1.5 macromolecular complexes in cardiomyocytes. Nevertheless, our myocyte data at least demonstrate that depolarization is not independent of repolarization.

## Conclusion

Overall, this study gives the first proof of concept that the K_V_4.3 protein directly impacts on Na_V_1.5 current. Future studies employing appropriate disease models should explore the potential electrophysiological implications in (patho)physiological conditions, including BrS associated with gain-of-function mutations in *KCND3*.

## Author contributions

Experimental design: VP, RW, FC, AV, and CR; Data acquisition: VP, RW, and AV; Analysis and interpretation of data: VP, RW, SC, AV, and CR; Drafting manuscript: VP, RW, AV, and CR; Editing manuscript, and approval: VP, RW, SC, FC, AV, and CR; Funding: CR.

### Conflict of interest statement

The authors declare that the research was conducted in the absence of any commercial or financial relationships that could be construed as a potential conflict of interest.

## References

[B1] Agullo-Pascual E. Lin X. Leo-Macias A. Zhang M. Liang F.-X. Li Z.. (2014). Super-resolution imaging reveals that loss of the C-terminus of connexin43 limits microtubule plus-end capture and Na_V_1.5 localization at the intercalated disc. Cardiovasc. Res. 104, 371–381. 10.1093/cvr/cvu195 25139742 PMC4296112

[B2] Balse E. Boycott H. E. (2017). Ion channel trafficking: control of ion channel density as a target for arrhythmias? Front. Physiol. 8:808. 10.3389/fphys.2017.00808 29089904 PMC5650974

[B3] Barry P. H. Lynch J. W. (1991). Liquid junction potentials and small cell effects in patch-clamp analysis. J. Membr. Biol. 121, 101–117. 10.1007/BF01870526 1715403

[B4] Benson D. W. Wang D. W. Dyment M. Knilans T. K. Fish F. A. Strieper M. J.. (2003). Congenital sick sinus syndrome caused by recessive mutations in the cardiac sodium channel gene (*SCN5A*). J. Clin. Invest. 112, 1019–1028. 10.1172/JCI18062 14523039 PMC198523

[B5] Berecki G. Wilders R. de Jonge B. van Ginneken A. C. G. Verkerk A. O. (2010). Re-evaluation of the action potential upstroke velocity as a measure of the Na^+^ current in cardiac myocytes at physiological conditions. PLoS ONE 5:e15772. 10.1371/journal.pone.0015772 21217835 PMC3013114

[B6] Bondarenko V. E. Szigeti G. P. Bett G. C. L. Kim S.-J. Rasmusson R. L. (2004). Computer model of action potential of mouse ventricular myocytes. Am. J. Physiol. Heart Circ. Physiol. 287, H1378–H1403. 10.1152/ajpheart.00185.2003 15142845

[B7] Brachet A. Leterrier C. Irondelle M. Fache M.-P. Racine V. Sibarita J.-B.. (2010). Ankyrin G restricts ion channel diffusion at the axonal initial segment before the establishment of the diffusion barrier. J. Cell. Biol. 191, 383–395. 10.1083/jcb.201003042 20956383 PMC2958486

[B8] Casini S. Verkerk A. O. van Borren M. M. G. J. van Ginneken A. C. G. Veldkamp M. W. de Bakker J. M. T.. (2009). Intracellular calcium modulation of voltage-gated sodium channels in ventricular myocytes. Cardiovasc. Res. 81, 72–81. 10.1093/cvr/cvn274 18829699

[B9] Chen Q. Kirsch G. E. Zhang D. Brugada R. Brugada J. Brugada P.. (1998). Genetic basis and molecular mechanism for idiopathic ventricular fibrillation. Nature 392, 293–296. 10.1038/32675 9521325

[B10] Coronel R. Casini S. Koopmann T. T. Wilms-Schopman F. J. G. Verkerk A. O. de Groot J. R.. (2005). Right ventricular fibrosis and conduction delay in a patient with clinical signs of Brugada syndrome: a combined electrophysiological, genetic, histopathologic, and computational study. Circulation 112, 2769–2777. 10.1161/CIRCULATIONAHA.105.532614 16267250

[B11] Crotti L. Marcou C. A. Tester D. J. Castelletti S. Giudicessi J. R. Torchio M.. (2012). Spectrum and prevalence of mutations involving BrS1- through BrS12-susceptibility genes in a cohort of unrelated patients referred for Brugada syndrome genetic testing: implications for genetic testing. J. Am. Coll. Cardiol. 60, 1410–1418. 10.1016/j.jacc.2012.04.037 22840528 PMC3624764

[B12] Cuellar A. A. Lloyd C. M. Nielsen P. F. Bullivant D. P. Nickerson D. P. Hunter P. J. (2003). An overview of CellML 1.1, a biological model description language. Simulation 79, 740–747. 10.1177/0037549703040939

[B13] Delpón E. Cordeiro J. M. Núñez L. Thomsen P. E. B. Guerchicoff A. Pollevick G. D.. (2008). Functional effects of *KCNE3* mutation and its role in the development of Brugada syndrome. Circ. Arrhythm. Electrophysiol. 1, 209–218. 10.1161/CIRCEP.107.748103 19122847 PMC2585750

[B14] Di Diego J. M. Cordeiro J. M. Goodrow R. J. Fish J. M. Zygmunt A. C. Pérez G. J.. (2002). Ionic and cellular basis for the predominance of the Brugada syndrome phenotype in males. Circulation 106, 2004–2011. 10.1161/01.CIR.0000032002.22105.7A 12370227

[B15] El Refaey M. M. Mohler P. J. (2017). Ankyrins and spectrins in cardiovascular biology and disease. Front. Physiol. 8:852. 10.3389/fphys.2017.00852 29163198 PMC5664424

[B16] Garny A. Kohl P. Noble D. (2003). Cellular open resource (COR): a public CellML based environment for modelling biological function. Int. J. Bifurcat. Chaos 13, 3579–3590. 10.1142/S021812740300882X

[B17] Gellens M. E. George A. L. Chen L. Q. Chahine M. Horn R. Barchi R. L.. (1992). Primary structure and functional expression of the human cardiac tetrodotoxin-insensitive voltage-dependent sodium channel. Proc. Natl. Acad. Sci. U.S.A. 89, 554–558. 10.1073/pnas.89.2.554 1309946 PMC48277

[B18] Giles W. R. van Ginneken A. C. G. (1985). A transient outward current in isolated cells from the crista terminalis of rabbit heart. J. Physiol. 368, 243–264. 10.1113/jphysiol.1985.sp015856 2416913 PMC1192595

[B19] Hoogendijk M. G. Opthof T. Postema P. G. Wilde A. A. M. de Bakker J. M. T. Coronel R. (2010a). The Brugada ECG pattern: a marker of channelopathy, structural heart disease, or neither? Toward a unifying mechanism of the Brugada syndrome. Circ. Arrhythm. Electrophysiol. 3, 283–290. 10.1161/CIRCEP.110.937029 20551422

[B20] Hoogendijk M. G. Potse M. Linnenbank A. C. Verkerk A. O. den Ruijter H. M. van Amersfoorth S. C. M.. (2010b). Mechanism of right precordial ST-segment elevation in structural heart disease: excitation failure by current-to-load mismatch. Heart Rhythm 7, 238–248. 10.1016/j.hrthm.2009.10.007 20022821

[B21] Hu D. Barajas-Martínez H. Medeiros-Domingo A. Crotti L. Veltmann C. Schimpf R. (2012). A novel rare variant in *SCN1Bb* linked to Brugada syndrome and SIDS by combined modulation of Na_V_1.5 and K_V_4.3 channel currents. Heart Rhythm 9, 760–769. 10.1016/j.hrthm.2011.12.006 22155597 PMC3334446

[B22] Kucera J. P. Rohr S. Kleber A. G. (2017). Microstructure, cell-to-cell coupling, and ion currents as determinants of electrical propagation and arrhythmogenesis. Circ. Arrhythm. Electrophysiol. 10:e004665. 10.1161/CIRCEP.117.004665 28912204

[B23] Le Scouarnec S. Karakachoff M. Gourraud J.-B. Lindenbaum P. Bonnaud S. Portero V.. (2015). Testing the burden of rare variation in arrhythmia-susceptibility genes provides new insights into molecular diagnosis for Brugada syndrome. Hum. Mol. Genet. 24, 2757–2763. 10.1093/hmg/ddv036 25650408

[B24] Leterrier C. Vacher H. Fache M.-P. d'Ortoli S. A. Castets F. Autillo-Touati A.. (2011). End-binding proteins EB3 and EB1 link microtubules to ankyrin G in the axon initial segment. Proc. Natl. Acad. Sci. U.S.A. 108, 8826–8831. 10.1073/pnas.1018671108 21551097 PMC3102358

[B25] Lieve K. V. Verkerk A. O. Podliesna S. van der Werf C. Tanck M. W. Hofman N.. (2017). Gain-of-function mutation in *SCN5A* causes ventricular arrhythmias and early onset atrial fibrillation. Int. J. Cardiol. 236, 187–193. 10.1016/j.ijcard.2017.01.113 28262340

[B26] Liu J. Kim K.-H. Morales M. J. Heximer S. P. Hui C.-C. Backx P. H. (2015). Kv4.3-encoded fast transient outward current is presented in Kv4.2 knockout mouse cardiomyocytes. PLoS ONE 10:e0133274. 10.1371/journal.pone.0133274 26196737 PMC4510596

[B27] Marsman R. F. J. Bezzina C. R. Freiberg F. Verkerk A. O. Adriaens M. E. Podliesna S.. (2014). Coxsackie and adenovirus receptor is a modifier of cardiac conduction and arrhythmia vulnerability in the setting of myocardial ischemia. J. Am. Coll. Cardiol. 63, 549–559. 10.1016/j.jacc.2013.10.062 24291282 PMC3926969

[B28] Matamoros M. Pérez-Hernández M. Guerrero-Serna G. Amorós I. Barana A. Núñez M.. (2016). Nav1.5 N-terminal domain binding to α1-syntrophin increases membrane density of human Kir2.1, Kir2.2 and Nav1.5 channels. Cardiovasc. Res. 110, 279–290. 10.1093/cvr/cvw009 26786162 PMC4836625

[B29] Milstein M. L. Musa H. Balbuena D. P. Anumonwo J. M. B. Auerbach D. S. Furspan P. B.. (2012). Dynamic reciprocity of sodium and potassium channel expression in a macromolecular complex controls cardiac excitability and arrhythmia. Proc. Natl. Acad. Sci. U.S.A. 109, E2134–E2143. 10.1073/pnas.1109370109 22509027 PMC3412015

[B30] Nademanee K. Veerakul G. Chandanamattha P. Chaothawee L. Ariyachaipanich A. Jirasirirojanakorn K.. (2011). Prevention of ventricular fibrillation episodes in Brugada syndrome by catheter ablation over the anterior right ventricular outflow tract epicardium. Circulation 123, 1270–1279. 10.1161/CIRCULATIONAHA.110.972612 21403098

[B31] Niwa N. Nerbonne J. M. (2010). Molecular determinants of cardiac transient outward potassium current (I_to_) expression and regulation. J. Mol. Cell. Cardiol. 48, 12–25. 10.1016/j.yjmcc.2009.07.013 19619557 PMC2813406

[B32] Niwa N. Wang W. Sha Q. Marionneau C. Nerbonne J. M. (2008). Kv4.3 is not required for the generation of functional I_to,f_ channels in adult mouse ventricles. J. Mol. Cell. Cardiol. 44, 95–104. 10.1016/j.yjmcc.2007.10.007 18045613 PMC2245858

[B33] Portero V. Le Scouarnec S. Es-Salah-Lamoureux Z. Burel S. Gourraud J.-B. Bonnaud S.. (2016). Dysfunction of the voltage-gated K^+^ channel β2 subunit in a familial case of Brugada syndrome. J. Am. Heart. Assoc. 5:e003122. 10.1161/JAHA.115.003122 27287695 PMC4937261

[B34] Remme C. A. Verkerk A. O. Nuyens D. van Ginneken A. C. G. van Brunschot S. Belterman C. N. W.. (2006). Overlap syndrome of cardiac sodium channel disease in mice carrying the equivalent mutation of human SCN5A-1795insD. Circulation 114, 2584–2594. 10.1161/CIRCULATIONAHA.106.653949 17145985

[B35] Rush S. Larsen H. (1978). A practical algorithm for solving dynamic membrane equations. IEEE Trans. Biomed. Eng. 25, 389–392. 10.1109/TBME.1978.326270 689699

[B36] Schott J. J. Alshinawi C. Kyndt F. Probst V. Hoorntje T. M. Hulsbeek M.. (1999). Cardiac conduction defects associate with mutations in SCN5A. Nat. Genet. 23, 20–21. 10.1038/12618 10471492

[B37] Shaw R. M. Rudy Y. (1997). Ionic mechanisms of propagation in cardiac tissue: roles of the sodium and L-type calcium currents during reduced excitability and decreased gap junction coupling. Circ. Res. 81, 727–741. 10.1161/01.RES.81.5.727 9351447

[B38] Ten Tusscher K. H. W. J. Panfilov A. V. (2006). Cell model for efficient simulation of wave propagation in human ventricular tissue under normal and pathological conditions. Phys. Med. Biol. 51, 6141–6156. 10.1088/0031-9155/51/23/014 17110776

[B39] Thomas S. P. Kucera J. P. Bircher-Lehmann L. Rudy Y. Saffitz J. E. Kléber A. G. (2003). Impulse propagation in synthetic strands of neonatal cardiac myocytes with genetically reduced levels of connexin43. Circ. Res. 92, 1209–1216. 10.1161/01.RES.0000074916.41221.EA 12730095 PMC2242733

[B40] Utrilla R. G. Nieto-Marín P. Alfayate S. Tinaquero D. Matamoros M. Pérez-Hernández M.. (2017). Kir2.1-Nav1.5 channel complexes are differently regulated than Kir2.1 and Nav1.5 channels alone. Front. Physiol. 8:903. 10.3389/fphys.2017.00903 29184507 PMC5694551

[B41] Vacher H. Yang J.-W. Cerda O. Autillo-Touati A. Dargent B. Trimmer J. S. (2011). Cdk-mediated phosphorylation of the Kvβ2 auxiliary subunit regulates Kv1 channel axonal targeting. J. Cell. Biol. 192, 813–824. 10.1083/jcb.201007113 21357749 PMC3051814

[B42] van Bemmelen M. X. van Rougier J.-S. Gavillet B. Apothéloz F. Daidié D. Tateyama M.. (2004). Cardiac voltage-gated sodium channel Na_V_1.5 is regulated by Nedd4-2 mediated ubiquitination. Circ. Res. 95, 284–291. 10.1161/01.RES.0000136816.05109.89 15217910

[B43] Veerman C. C. Podliesna S. Tadros R. Lodder E. M. Mengarelli I. de Jonge B. (2017). The Brugada syndrome susceptibility gene *HEY2* modulates cardiac transmural ion channel patterning and electrical heterogeneity. Circ. Res. 121, 537–548. 10.1161/CIRCRESAHA.117.310959 28637782

[B44] Verkerk A. O. Veerman C. C. Zegers J. G. Wilders R. (2016). Effects of the transient outward potassium current on action potential upstroke velocities tested using the dynamic clamp technique. Comput. Cardiol. 43, 257–260. 10.22489/CinC.2016.076-143

[B45] Wilde A. A. M. Postema P. G. Di Diego J. M. Viskin S. Morita H. Fish J. M.. (2010). The pathophysiological mechanism underlying Brugada syndrome: depolarization versus repolarization. J. Mol. Cell. Cardiol. 49, 543–553. 10.1016/j.yjmcc.2010.07.012 20659475 PMC2932806

[B46] Wilders R. (2012). Arrhythmogenic right ventricular cardiomyopathy: considerations from *in silico* experiments. Front. Physiol. 3:168. 10.3389/fphys.2012.00168 22754532 PMC3385583

[B47] You T. Mao W. Cai B. Li F. Xu H. (2015). Two novel Brugada syndrome-associated mutations increase K_V_4.3 membrane expression and function. Int. J. Mol. Med. 36, 309–315. 10.3892/ijmm.2015.2223 26016905 PMC4494594

[B48] Zhao X.-J. Zhu C. Tian L.-Y. Fu Y.-C. Zhang Y. Chen X.. (2017). Kv4.3 modulates the distribution of hERG. Sci. Rep. 7:17757. 10.1038/s41598-017-17837-6 29259226 PMC5736654

