# Supplementary material for: KV4.3 Expression Modulates NaV1.5 Sodium Current
Source: Front Physiol. 2018 Mar 12;9:178. doi: 10.3389/fphys.2018.00178 (PMC5857579; doi:10.3389/fphys.2018.00178)
Supplement: Supplementary file 1 [file DataSheet1.pdf]

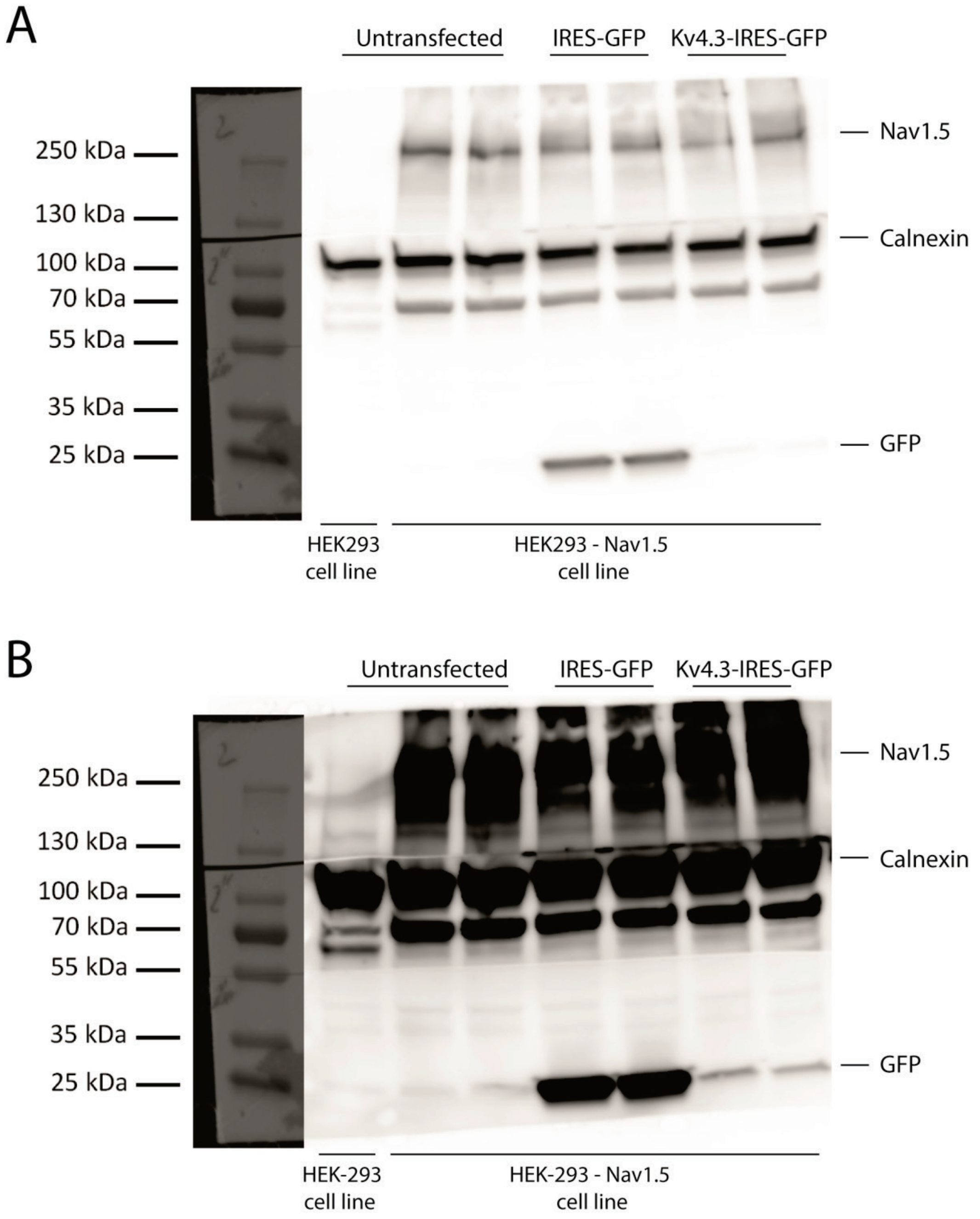

Figure S1. Original image of the immunoblot represented in Figure 3 and typical example of immunoblot obtained. Typical result of chemiluminescence acquisition obtained after **(A)** 1 minute exposure and **(B)** 15 minutes exposure. Na_V1.5 and calnexin signal were extracted from the image obtained after 1 minute exposure. The GFP signal represented in Figure 3 was extracted from the image after 15 minutes exposure.

Table S1. Biophysical properties of $Na_V1.5$ and $K_V4.3$ currents recorded in the HEK293-$Na_V1.5$ cell line after transfection of the IRES-GFP or *KCND3*-IRES-GFP plasmid.

	IRES-GFP	*n*	$K_V4.3$-IRES-GFP	*n*
I_{Na}				
Current density (pA/pF)	−609 ± 62	15	−447 ± 61*[a]	19
Activation				
$V_{½}$ (mV)	−45.9 ± 1.4	15	−45.15 ± 0.9	19
k (mV)	7.5 ± 0.4	15	7.0 ± 0.3	19
Inactivation				
$V_{½}$ (mV)	−91.6 ± 1.2	15	−91.3 ± 1	19
k (mV)	−6.15 ± 0.17	15	−5.7 ± 0.2	19
Time course of inactivation				
$t_{50\%}$ (ms)	0.88 ± 0.06	15	0.84 ± 0.03	19
I_{to}				
Current density (pA/pF)	23.2 ± 6.8	10	438 ± 65.9*[b]	8
Activation				
$V_{½}$ (mV)	—		−45.9 ± 2.6	8
k (mV)	—		−5.6 ± 0.4	8
Recovery from inactivation				
τ (ms)	—		160 ± 29	4

*$P < 0.05$. [a]Current density at −40 mV ($P = 0.027$). [b]Current density at +40 mV ($P < 0.001$). The time course of inactivation for I_{Na} was characterized through its time required for 50% of current decay at −30 mV ($t_{50\%}$). For the $Na_V1.5$ and $K_V4.3$ current densities, which were determined at multiple values of membrane potential (cf. Figures 1B and 2B), a two-way repeated measures ANOVA followed by pairwise comparison using the Student-Newman-Keuls test was performed. The other biophysical properties were compared using a Student's *t*-test.

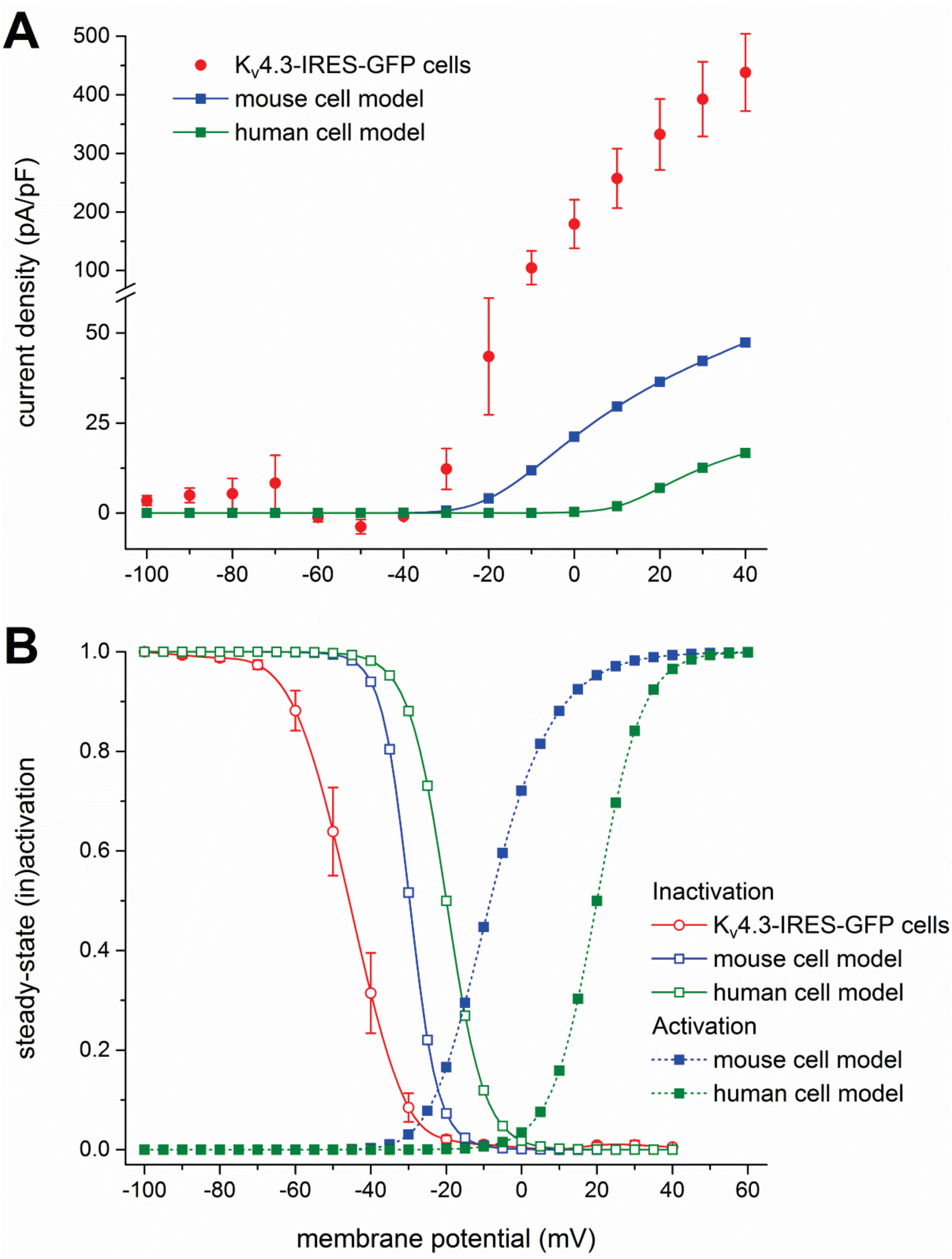

Figure S2. Characteristics of the experimentally studied K_V4.3 current and the transient outward current in ventricular cell models. (A) Peak current-voltage relationship of the K_V4.3 current in HEK293-Na_V1.5 cells transfected with *KCND3* (red circles; mean±SEM, n = 8), the transient outward current in the apical version of the mouse ventricular cell model by Bondarenko et al. (2004) (blue squares), and the transient outward current in the human ventricular cell model by Ten Tusscher and Panfilov (2006) (green squares). **(B)** Associated steady-state activation and inactivation curves.

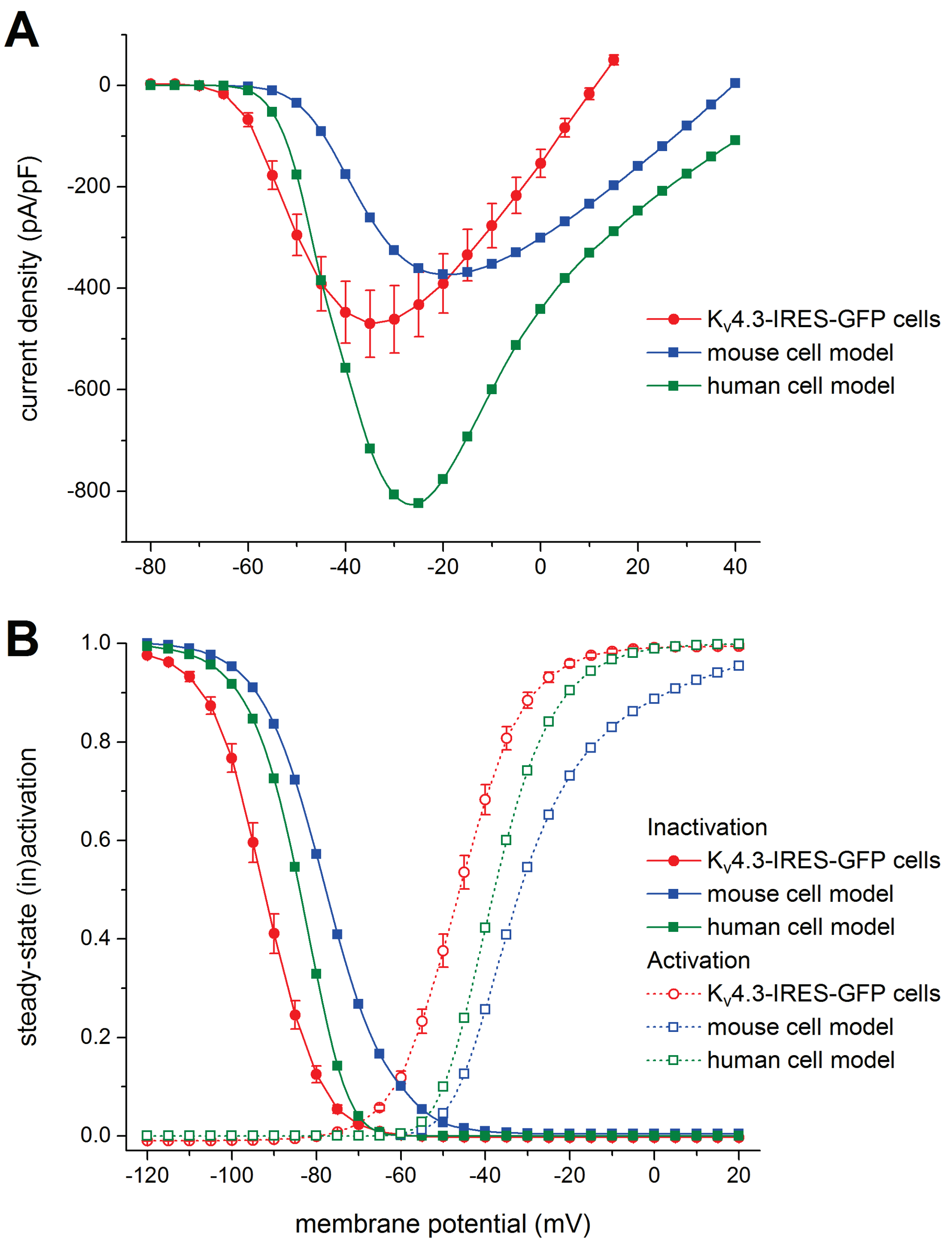

Figure S3. Characteristics of the experimentally studied Na_V1.5 current and the fast sodium current in ventricular cell models. (A) Peak current-voltage relationship of the Na_V1.5 current in HEK293-Na_V1.5 cells transfected with *KCND3* (red circles; mean±SEM, *n* = 19), the fast sodium current in the apical version of the mouse ventricular cell model by Bondarenko et al. (2004) (blue squares), and the fast sodium current current in the human ventricular cell model by Ten Tusscher and Panfilov (2006) (green squares). **(B)** Associated steady-state activation and inactivation curves. Note that the experimental data were obtained at room temperature and an extracellular sodium concentration of 20 mM.

References

Bondarenko, V. E., Szigeti, G. P., Bett, G. C. L., Kim, S.-J., and Rasmusson, R. L. (2004). Computer model of action potential of mouse ventricular myocytes. *Am. J. Physiol. Heart Circ. Physiol.* 287, H1378–H1403. doi: 10.1152/ajpheart.00185.2003

Ten Tusscher, K. H. W. J., and Panfilov, A. V. (2006). Cell model for efficient simulation of wave propagation in human ventricular tissue under normal and pathological conditions. *Phys. Med. Biol.* 51, 6141–6156. doi: 10.1088/0031-9155/51/23/014
